# Filtrations on Springer fiber cohomology and Kostka polynomials

**DOI:** 10.1007/s11005-017-1002-7

**Published:** 2017-09-26

**Authors:** Gwyn Bellamy, Travis Schedler

**Affiliations:** 1 School of Mathematics and Statistics, University of Glasgow, University Gardens, Glasgow, G12 8QW UK; 2 Department of Mathematics, Imperial College London, South Kensington Campus, London, SW7 2AZ UK

**Keywords:** Equivariant D-modules, Kostka polynomials, Poisson-de Rham homology, W-algebras, Springer fibers, Nilpotent cone, Harish-Chandra homomorphism, Grothendieck–Springer resolution, 17B63, 14F10, 14M15

## Abstract

We prove a conjecture which expresses the bigraded Poisson-de Rham homology of the nilpotent cone of a semisimple Lie algebra in terms of the generalized (one-variable) Kostka polynomials, via a formula suggested by Lusztig. This allows us to construct a canonical family of filtrations on the flag variety cohomology, and hence on irreducible representations of the Weyl group, whose Hilbert series are given by the generalized Kostka polynomials. We deduce consequences for the cohomology of all Springer fibers. In particular, this computes the grading on the zeroth Poisson homology of all classical finite W-algebras, as well as the filtration on the zeroth Hochschild homology of all quantum finite W-algebras, and we generalize to all homology degrees. As a consequence, we deduce a conjecture of Proudfoot on symplectic duality, relating in type A the Poisson homology of Slodowy slices to the intersection cohomology of nilpotent orbit closures. In the last section, we give an analogue of our main theorem in the setting of mirabolic D-modules.

## Introduction

Let $\mathfrak {g}$ be a semisimple complex Lie algebra, $\mathcal {N}\subseteq \mathfrak {g}^*$ the nilpotent cone (of elements whose coadjoint orbit is stable under dilations), *W* the Weyl group, and *G* a simply connected complex Lie group with ${\text {Lie}}G = \mathfrak {g}$. The Springer correspondence associates to every irreducible representation $\chi$ of *W* a pair of a nilpotent coadjoint orbit $\mathcal {O}_\chi \subseteq \mathfrak {g}^*$ and a local system $L_\chi$ on $\mathcal {O}_\chi$. Let $\mathcal {B}$ be the flag variety and $\rho : T^* \mathcal {B} \rightarrow \mathcal {N}$ the Springer resolution. Then the cohomology of $T^* \mathcal {B}$, or equivalently of $\mathcal {B}$, is endowed by the Springer correspondence with a *W*-action. The graded multiplicity space of each irreducible representation $\chi$ of *W* has Hilbert series given by the generalized Kostka polynomial $K_{\mathfrak {g},\chi }(t)$, which in the case of $\mathfrak {g}=\mathfrak {sl}_n$ is an ordinary one-variable Kostka polynomial. Precisely, we set $K_{\mathfrak {g},\chi }(t) := \sum _{i \ge 0} t^i\dim {\text {Hom}}_W(\chi , H^{2\dim \mathcal {B}- 2i}(\mathcal {B}, \mathbf {C}))$, where $\dim$ always refers to the complex dimension. Note that, as a graded *W*-module, $H^*(\mathcal {B},\mathbf {C}) \cong {\text {Sym}}\,\mathfrak {h}/ ({\text {Sym}}\,\mathfrak {h})_+^W)$, putting $\mathfrak {h}$ in degree two, with $(({\text {Sym}}\,\mathfrak {h})_+^W)$ the ideal generated by the positive-degree *W*-invariant elements of ${\text {Sym}}\,\mathfrak {h}$.

By a theorem of [6], which was conjectured in [8, Conjecture 1.21.(b)] to generalize to arbitrary symplectic resolutions, $H^*(T^*\mathcal {B}) \cong {\text {HP}}_{\dim \mathcal {N}-*}^{DR}(\mathcal {N})$, where the latter is the Poisson-de Rham homology of $\mathcal {N}$, defined in [5]. Briefly, the Poisson-de Rham homology of a Poisson variety *Y* is defined as the derived pushforward ${\text {HP}}_i^{DR}(Y) := H^{-i} \pi _* M(Y)$ of the $\mathcal {D}$-module *M*(*Y*) on *Y* to a point, where *M*(*Y*) is defined by the property ${\text {Hom}}(M(Y), N) = \Gamma _{\mathcal {D}}(Y,N)^{H(Y)}$ for *H*(*Y*) the Lie algebra of Hamiltonian vector fields on *Y*, *N* an arbitrary $\mathcal {D}$-module on *Y* (in the sense of Kashiwara), and $\Gamma _{\mathcal {D}}(Y,N)$ the global sections of *N*: see Remark 4.1 or [4, 5] for details. In the case of the nilpotent cone, the Poisson-de Rham homology does not see the *W*-action, since *W* does not act on $\mathcal {N}$, unlike on the cohomology of $T^*\mathcal {B}$. On the other hand, $\mathcal {N}$ has a dilation action which endows ${\text {HP}}_*^{DR}(\mathcal {N})$ with a second grading, which is not seen in $H^*(T^*\mathcal {B})$. It is interesting to compute this grading. Moreover, this difference makes it clear that the isomorphism of [5] cannot be canonical, and it is interesting to correct this deficiency.

Lusztig suggested a simple formula for this bigrading ([19, Conjecture 8.1]):

1.1 $$\begin{aligned} h({\text {HP}}_*^{DR}(\mathcal {N});x,y)=\sum _{\chi \in {\text {Irrep}}(W)} K_{\mathfrak {g},\chi }(x^2) K_{\mathfrak {g},\chi }(y^{-2}). \end{aligned}$$

In this paper we prove this conjecture, in the following stronger form, as a simple application of a theorem of Hotta and Kashiwara. Let $\sigma$ denote the sign representation of *W*.

### Theorem 1.1

There is a canonical isomorphism of bigraded vector spaces

$$\begin{aligned} {\text {HP}}_*^{DR}(\mathcal {N}) \cong {\text {Hom}}_W({\text {Sym}}\,\mathfrak {h}/({\text {Sym}}\,\mathfrak {h})_+^W) \otimes \sigma , H^{2 \dim \mathcal {B}-*}(T^*\mathcal {B})). \end{aligned}$$

Here the weight grading on the LHS corresponds to the grading on ${\text {Sym}}\,\mathfrak {h}/(({\text {Sym}}\,\mathfrak {h})^W_+)$ on the RHS (with $\mathfrak {h}$ in degree two), and the second grading is by the asterisk $*$. This isomorphism accomplishes our goal of producing a canonical isomorphism.

### Remark 1.2

Using the homotopy equivalence $T^*\mathcal {B} \simeq \mathcal {B}$ together with Poincaré duality for $\mathcal {B}$, we can rewrite the theorem more simply as ${\text {HP}}_*^{DR}(\mathcal {N}) \cong {\text {Hom}}_W({\text {Sym}}\,\mathfrak {h}/(({\text {Sym}}\,\mathfrak {h})_+^W), H^{*}(\mathcal {B}))$, but the way it is written is more natural; for example, the aforementioned general conjecture states ${\text {HP}}_*^{DR}(X) \cong H^{\dim X - *}(\widetilde{X})$ for symplectic resolutions $\widetilde{X} \rightarrow X$.

We go further and produce canonical *filtrations* on the cohomology of the flag variety whose Hilbert series is given in (1.1):

### Theorem 1.3

For every element $\lambda \in \mathfrak {h}_{{\text {reg}}}^*$, there is a canonical associated filtration $\mathcal {F}_\lambda$ on $H^{2 \dim \mathcal {B}-*}(T^*\mathcal {B})$ whose associated graded vector space is ${\text {HP}}_*^{DR}(\mathcal {N})$. This is *W*-equivariant: $\mathcal {F}_{w(\lambda )} = w(\mathcal {F}_\lambda )$.

The filtration is compatible with the cohomological grading; hence, the associated graded vector space is bigraded. As a result we obtain canonical filtrations on irreducible representations of Weyl groups.

### Corollary 1.4

To every $\lambda \in \mathfrak {h}_{{\text {reg}}}^*$, there is associated a canonical filtration on every irreducible representation $\chi$ of *W* whose associated graded vector space has Hilbert series $K_{\mathfrak {g},\chi }(y^{-2})$.

This recovers in particular the noncanonical isomorphism predicted in [19, Conjecture 8.1].

### Example 1.5

Let $\mathfrak {g}=\mathfrak {sl}_n$ and let $\chi = \mathfrak {h}^* \cong \mathbf {C}^{n-1}$ be the (dual) reflection representation. Consider $\chi$ to be (in coordinates) $\mathbf {C}^n / \mathbf {C}\cdot (1,1,\ldots ,1)$. Let $\lambda \in \chi$ be the image of $(a_1, \ldots , a_n) \in \mathbf {C}^n$. Then the resulting filtration on $\chi$, which we call the Vandermonde filtration, is given as follows: for every $0 \le i \le n$, $F^{2i-2\dim \mathcal {B}}(\chi )$ is the span of $(a_1^j, \ldots , a_n^j)$ for $1 \le j \le i$.

As we observe, the construction of Corollary 1.4 actually generalizes from Weyl groups to arbitrary complex reflection groups. We will study the resulting filtrations in detail in future work.

We deduce many consequences and extensions of the above results to Slodowy slices, *W*-algebras, and Springer fibers. In more detail, let $\phi \in \mathcal {N}$ be any point. Then one can consider the Slodowy slice $S_\phi \cap \mathcal {N}$ in $\mathcal {N}$ to the coadjoint orbit $\mathcal {O}_\phi := G \cdot \phi \subseteq \mathcal {N}$. (We recall its construction in Sect. 2). The ring of functions $\mathcal {O}(S_\phi \cap \mathcal {N})$ is also called a (centrally reduced) classical W-algebra.

The above results allow us to deduce the grading on the zeroth Poisson homology,

$$\begin{aligned} {\text {HP}}_0(\mathcal {O}(S_\phi \cap \mathcal {N})):=\mathcal {O}(S_\phi \cap \mathcal {N})/\{\mathcal {O}(S_\phi \cap \mathcal {N}),\mathcal {O}(S_\phi \cap \mathcal {N})\} = {\text {HP}}_0^{DR}(S_\phi \cap \mathcal {N}), \end{aligned}$$

as well as the filtration on the quantizations of $S_\phi \cap \mathcal {N}$, which are (centrally reduced) quantum W-algebras. Geometrically, these naturally assign to the top cohomology of each Springer fiber $\rho ^{-1}(\phi )$ a $\mathfrak {h}_{{\text {reg}}}^*$-family of filtrations whose Hilbert series we compute.

As a consequence, when $\mathfrak {g}=\mathfrak {sl}_n$, and hence $Y=S_\phi \cap \mathcal {N}$ is *symplectically dual* to a corresponding coadjoint orbit $Y^! \subseteq \mathcal {N}$, we prove a case of a conjecture of Proudfoot, which states (for general symplectic dual cones *Y* and $Y^!$) that ${\text {HP}}_0(\mathcal {O}(Y)) \cong {\text {IH}}^*(Y^!)$ as graded vector spaces, with ${\text {IH}}^*(Y^!)$ the intersection cohomology of $Y^!$.

We also give formulas for the higher Poisson-de Rham homology of Slodowy slices and for the zeroth Hochschild homology of their quantizations by finite *W*-algebras.

### Remark 1.6

Note that (1.1) implies that the weight grading on ${\text {HP}}^{DR}_*(\mathcal {N})$ is nonpositive. This is somewhat unusual; for example, whenever the zeroth Poisson homology of a conical Poisson variety is at least two-dimensional it will have (some) positive weights, as will happen for many Slodowy slices in the nilpotent cone, cf. Corollary 2.5. Note that, for varieties admitting a symplectic resolution for which [8, Conjecture 1.3.(b)] holds, this condition that the zeroth Poisson homology has dimension at least two is equivalent to the statement that the fiber over the vertex in the symplectic resolution has multiple Lagrangian components. (In particular, it is not irreducible.)

The proofs, given in Sect. 4, involve a study of the Harish-Chandra $\mathcal {D}$-module on $\mathcal {N}$, following Hotta and Kashiwara in [12]. In Sect. 6.2, we give a generalization of our main result to the setting of mirabolic $\mathcal {D}$-modules, which computes the weakly equivariant structure of the mirabolic Harish-Chandra $\mathcal {D}$-module on $\mathfrak {gl}_n \times \mathbf {C}^n$, defined in [9].

We begin the body of the paper in Sect. 2 with a detailed statement of our results on the grading associated with the cohomology of Springer fibers as well as to the Poisson and Hochschild homology of W-algebras. The application to Proudfoot’s conjecture on symplectic duality is then given in Sect. 3. In the remaining sections, we prove our results using $\mathcal {D}$-modules, recalling first some of the necessary background. In the last section, we explain an alternative proof of Lusztig’s formula using Hamiltonian reduction. We then use this to generalize this result to the mirabolic setting, i.e., the setting of ${\mathsf {SL}}_n$-equivariant $\mathcal {D}$-modules on $\mathfrak {sl}_n \times \mathbf {C}^n$.

## Springer fibers and *W*-algebras

Let $\phi \in \mathcal {N}$. We may then consider the Springer fiber $\rho ^{-1}(\phi ) \subseteq T^* \mathcal {B}$, which reduces to $\mathcal {B}$ itself in the case $\phi =0$.

There is a beautiful construction of a transverse slice, called the Kostant–Slodowy slice, which we denote $S_\phi$, to $\mathcal {O}_\phi$ in $\mathfrak {g}^*$, which is an affine linear space defined as follows: Let $\langle -,-\rangle$ be a nondegenerate invariant bilinear form on $\mathfrak {g}$ (e.g., the Killing form) and $\varPhi : \mathfrak {g}\rightarrow \mathfrak {g}^*$ be the resulting isomorphism. Then $e:=\varPhi ^{-1}(\phi )$ is ad-nilpotent. The Jacobson–Morozov theorem states that the element *e* can be extended (nonuniquely) to a so-called $\mathfrak {sl}_2$ triple (*e*, *h*, *f*) of elements of $\mathfrak {g}$ satisfying the relations $[h,e]=2e, [h,f]=-2f$, and $[e,f]=h$. Then $S_\phi$ can be defined as $\varPhi (e + \ker ({\text {ad}}f))$. Moreover, Kazhdan defined a canonical contracting $\mathbf {C}^\times$ action on $S_\phi$ to $\phi$, by $\lambda \cdot \phi = \lambda ^{2-{\text {ad}}(h)^*}(\phi )$; the induced grading on $\mathcal {O}(S_\phi )$ is called the Kazhdan grading. Using this action, the resolution $\rho$ restricts to a $\mathbf {C}^\times$-equivariant symplectic resolution $\rho : \rho ^{-1}(S_\phi \cap \mathcal {N}) \rightarrow S_\phi \cap \mathcal {N}$, which topologically contracts to the fixed locus $\rho ^{-1}(\phi ) \rightarrow \{\phi \}$. Then, as before, [8, Conjecture 1.3.(b)] states in this case that $H^*(\rho ^{-1}(S_\phi \cap \mathcal {N})) \cong {\text {HP}}^{DR}_{\dim S_\phi \cap \mathcal {N}- *}(S_\phi \cap \mathcal {N})$, which was proved in [6, Theorem 1.13]. Since the Poisson-de Rham homology is bigraded by homological and Kazhdan gradings, this yields a bigrading on the cohomology of the Springer fiber $H^*(\rho ^{-1}(\phi ))$, which was not studied in [6]. Our goal is to compute this grading.

For now, we describe the grading in top degree, $H^{\dim \rho ^{-1}(\phi )}(\rho ^{-1}(\phi ))$ (see Corollary 2.5 for the general formula in terms of intersection cohomology). This has an explicit algebraic interpretation in terms of *W*-algebras. Namely, the finite *W*-algebra $W_\phi$ is the coordinate ring of $S_\phi$, and its central reduction $W_\phi ^0$ is the coordinate ring of $S_\phi \cap \mathcal {N}$. Let us recall their explicit algebraic description, along with the Poisson structure, following [10] and [13].

Since ${\text {ad}}(h)$ is semisimple, we get a decomposition $\mathfrak {g}= \bigoplus _i \mathfrak {g}_i$ where $\mathfrak {g}_i$ is the eigenspace of ${\text {ad}}(h)$ of eigenvalue *i*. Equip $\mathfrak {g}$ with the skew-symmetric form $\omega _\phi (x,y) := \phi ([x,y]) = \langle e, [x, y] \rangle$. This restricts to a nondegenerate pairing on $\mathfrak {g}_{-1}$. Fix a Lagrangian subspace $\mathfrak {l} \subseteq \mathfrak {g}_{-1}$. Then we define

2.1 $$\begin{aligned} \mathfrak {m}_\phi := \mathfrak {l} \oplus \bigoplus _{i \le -2} \mathfrak {g}_i. \end{aligned}$$

We also define the shift

2.2 $$\begin{aligned} \mathfrak {m}_\phi ' := \{x-\phi (x) \mid x \in \mathfrak {m}_\phi \} \subseteq {\text {Sym}}\,\mathfrak {g}. \end{aligned}$$

The *W*-algebra $W_\phi$ is then defined by

2.3 $$\begin{aligned} W_\phi = ({\text {Sym}}\,\mathfrak {g}/ \mathfrak {m}_\phi ' {\text {Sym}}\,\mathfrak {g})^{\mathfrak {m}_\phi }. \end{aligned}$$

In other words, this is the Hamiltonian reduction of $\mathfrak {g}^*$ with respect to the Lie algebra $\mathfrak {m}_\phi$ and its character $\phi$. By construction, $W_\phi$ is a Poisson algebra (with respect to the Poisson bracket induced from ${\text {Sym}}\,\mathfrak {g}$). In more detail, if $x +\mathfrak {m}_\phi ' {\text {Sym}}\,\mathfrak {g}\in W_\phi$, then $\{x, \mathfrak {m}_\phi \} \subseteq \mathfrak {m}_\phi ' {\text {Sym}}\,\mathfrak {g}$, and hence $\{x, \mathfrak {m}_\phi ' {\text {Sym}}\,\mathfrak {g}\} \subseteq \mathfrak {m}_\phi ' {\text {Sym}}\,\mathfrak {g}$. Thus, the Poisson bracket on ${\text {Sym}}\,\mathfrak {g}$ induces one on $W_\phi$.

Given any central character $\eta : Z({\text {Sym}}\,\mathfrak {g}) = ({\text {Sym}}\,\mathfrak {g})^{\mathfrak {g}} \rightarrow \mathbf {C}$, we can form the central reduction $W_\phi ^\eta := W_\phi / \ker (\eta ) W_\phi$. Here $Z({\text {Sym}}\,\mathfrak {g})$ denotes the Poisson center of ${\text {Sym}}\,\mathfrak {g}$. Then, $W_\phi$ and $W_\phi ^0$ are the coordinate rings of the Kostant–Slodowy slices:

$$\begin{aligned} W_\phi = \mathcal {O}(S_\phi ), \quad W_\phi ^0 = \mathcal {O}(S_\phi \cap \mathcal {N}). \end{aligned}$$

Note that, for general $\eta$, ${\mathsf {Spec}}W_\phi ^\eta$ is a deformation of $S_\phi \cap \mathcal {N}$, namely the Kostant–Slodowy slice $S_\phi$ intersected with the closure of the regular coadjoint orbit on which every $f \in ({\text {Sym}}\,\mathfrak {g})^\mathfrak {g}$ restricts to the constant function $\eta (f)$. Moreover $W_\phi ^\eta$ is a filtered algebra with ${\text {gr}}W_\phi ^{\eta } = W_\phi ^0$.

Recall that the *zeroth Poisson homology* of a Poisson algebra *A* is ${\text {HP}}_0(A) := A/\{A,A\}$. Then, as a corollary of our main theorem, we compute the graded structure of the zeroth Poisson homology of $W_\phi$ and $W_\phi ^0$, as well as the filtered structure of $W_\phi ^\eta$. We will need to use the Springer correspondence, which assigns (injectively) to each irreducible representation $\chi \in {\text {Irrep}}(W)$ the pair of a nilpotent coadjoint orbit $\mathcal {O}_\chi \subseteq \mathcal {N}$ and an irreducible local system $L_\chi$ on $\mathcal {O}_\chi$ [see the beginning of Sect. 4 for an explicit definition of $(\mathcal {O}_\chi ,L_\chi )$]. Define the following polynomial (cf. [19, (8.2)]):

2.4 $$\begin{aligned} P_{\phi }(y) := y^{\dim \mathcal {O}_{\phi }} \sum _{\chi \in {\text {Irrep}}(W) \mid \mathcal {O}_\chi =\mathcal {O}_\phi } {\text {rk}}L_\chi \cdot K_{\mathfrak {g},\chi }(y^{-2}). \end{aligned}$$

### Corollary 2.1

The Hilbert series of ${\text {HP}}_0(W^0_\phi )$, as well as of ${\text {gr}}{\text {HP}}_0(W^\eta _\phi )$ for all $\eta$, is $P_\phi (y)$. Moreover, ${\text {HP}}_0(W_\phi )$ is freely generated over $({\text {Sym}}\,\mathfrak {g})^\mathfrak {g}$ generated by a graded vector space of the same Hilbert series.

Note that $W_\phi$ and hence ${\text {HP}}_0(W_\phi )$ inherit actions of ${\text {Stab}}_G(e,h,f)$ which commute with the dilation action and hence preserve degrees; on ${\text {HP}}_0(W_\phi )$ this action factors through the finite group $\pi _0 {\text {Stab}}_G(e,h,f)$ since the Lie algebra of ${\text {Stab}}_G(e,h,f) \subseteq G$ acts trivially. As observed in [6], $\pi _0 {\text {Stab}}_G(e,h,f) = \pi _0 {\text {Stab}}_G(e)$ (since ${\text {Stab}}_G(e,h,f)$ is the reductive part of ${\text {Stab}}_G(e)$), which is obviously equal to $\pi _0 {\text {Stab}}_G(\phi )$, and the isomorphism $H^*(\rho ^{-1}(S_\phi \cap \mathcal {N})) \cong {\text {HP}}^{DR}_{\dim S_\phi \cap \mathcal {N}- *}(S_\phi \cap \mathcal {N})$ is compatible with the $\pi _0 {\text {Stab}}_G(\phi )$-actions.

Moreover, $\pi _0 {\text {Stab}}_G(\phi ) = \pi _1 \mathcal {O}_\phi$ (since we assumed *G* simply connected), and for $\chi \in {\text {Irrep}}(W)$ such that $\mathcal {O}_\chi =\mathcal {O}_\phi$, the local system $L_\chi$ is an irreducible representation of $\pi _1 \mathcal {O}_\phi$. Thus, using the $\pi _0 {\text {Stab}}_G(\phi )$ action, we can refine the corollary to yield the following. Let $V_\chi ^*$ be a graded vector space with Hilbert series $K_{\mathfrak {g},\chi }(y^{-2})$.

### Corollary 2.2

As graded representations of $\pi _0 {\text {Stab}}_G(\phi )=\pi _1 \mathcal {O}_\phi$,

$$\begin{aligned} {\text {HP}}_0(W^0_\phi ) \cong \bigoplus _{\chi \in {\text {Irrep}}(W) \mid \mathcal {O}_\chi =\mathcal {O}_\phi } L_\chi \otimes V_\chi ^*[-\dim \mathcal {O}_\phi ]. \end{aligned}$$

Finally, for $W_\phi$ itself, Corollary 2.1 yields

2.5 $$\begin{aligned} h({\text {HP}}_0(W_\phi );y)=P_\phi (y) \prod _{i=1}^{r} (1-y^{2d_i})^{-1}, \end{aligned}$$

where *r* is the semisimple rank of $\mathfrak {g}$ and $d_1, \ldots , d_r$ are the degrees of the fundamental invariants (i.e., one-half the polynomial degrees of generators of $({\text {Sym}}\,\mathfrak {g})^\mathfrak {g}\cong ({\text {Sym}}\,\mathfrak {h})^W$, for $\mathfrak {h}\subseteq \mathfrak {g}$ a Cartan subalgebra with Weyl group *W*). Similarly, Corollary 2.2 implies we can write, as graded representations of $\pi _0 {\text {Stab}}_G(\phi )$,

2.6 $$\begin{aligned} {\text {HP}}_0(W_\phi ) \cong ({\text {Sym}}\,\mathfrak {g})^\mathfrak {g}\otimes \left( \bigoplus _{\chi \in {\text {Irrep}}(W) \mid \mathcal {O}_\chi =\mathcal {O}_\phi } L_\chi \otimes V_\chi ^*[-\dim \mathcal {O}_\phi ]\right) . \end{aligned}$$

### Hochschild cohomology of quantum W-algebras

Parallel to the previous corollaries, we can consider the quantum analogue of $W_\phi$, defined as:

2.7 $$\begin{aligned} W_\phi ^q := (U \mathfrak {g}/ \mathfrak {m}_\phi ' U \mathfrak {g})^{\mathfrak {m}_\phi }, \quad W_\phi ^{q,\eta } := W_\phi ^q / \ker (\eta ), \end{aligned}$$

where $\eta$ is a character of $Z(U\mathfrak {g}) \cong ({\text {Sym}}\,\mathfrak {g})^\mathfrak {g}$. By [6, Theorem 1.10.(i)], ${\text {gr}}{\text {HH}}_0(W^{q,\eta }_\phi ) \cong {\text {HP}}_0(W^0_\phi )$, and it follows that ${\text {gr}}{\text {HH}}_0(W^q_\phi ) \cong {\text {HP}}_0(W_\phi )$. Thus, the following is an immediate consequence of Corollary 2.1 and its proof is omitted:

#### Corollary 2.3

The Hilbert series of ${\text {gr}}{\text {HH}}_0(W^{q,\eta }_\phi )$, for all $\eta$, is $P_\phi (y)$. Moreover, ${\text {HH}}_0(W^q_\phi )$ is a free filtered module over $Z(U \mathfrak {g})$ generated by a filtered vector space whose associated graded vector space has Hilbert series $P_\phi (y)$.

Similarly, the associated graded vector space of ${\text {HH}}_0(W^q_\phi )$ has Hilbert series

2.8 $$\begin{aligned} h({\text {gr}}{\text {HH}}_0(W^q_\phi ); y)=P_\phi (y) \prod _{i=1}^{r} (1-y^{2d_i})^{-1}. \end{aligned}$$

#### Remark 2.4

As in Remark 2, ${\text {HH}}_0(W^{q,\eta }_\phi )$ and ${\text {HH}}_0(W^q_\phi )$ are also representations of the finite group $\pi _0 {\text {Stab}}_G(e,h,f)=\pi _0 {\text {Stab}}_G(\phi )$, and Corollaries 2.2 and (2.6) carry over replacing the left hand sides by ${\text {HH}}_0(W^{q,\eta }_\phi )$ and ${\text {HH}}_0(W^q_\phi )$, respectively, now viewing $V_\chi ^*$ as a filtered vector space.

### Higher cohomology of the Springer fiber

The next result describes the bigrading on the (full) cohomology of the Springer fiber, which is analogous to the associated graded vector space of $H^*(T^*\mathcal {B})$ appearing in Theorem 1.3. That is, we compute the Poisson-de Rham homology of the Slodowy slices to the nilpotent cone. We do not attempt here to construct actual filtrations on the Springer fiber cohomology.

When $\overline{\mathcal {O}_\chi } \supseteq \mathcal {O}_\phi$, we will consider the varieties (called S3 varieties, after Slodowy, Spaltenstein, and Springer), $S_{\chi ,\phi } := \overline{\mathcal {O}_\chi } \cap S_\phi$. Let ${\text {ih}}_{\chi ,\phi }(x) := h({\text {IH}}^*(S_{\chi ,\phi },L_\chi |_{\mathcal {O}_\chi \cap S_\phi });x)$ be the intersection cohomology Poincaré polynomial of $S_{\chi ,\phi }$ equipped with the local system $L_{\chi }|_{\mathcal {O}_\chi \cap S_\phi }$. For example, in the case $\mathfrak {g}=\mathfrak {sl}_n$, the $L_\chi$ are all trivial, and by [16, Theorem 2], we have ${\text {ih}}_{\chi ,\phi }(x)=x^{\dim S_{\chi ,\phi }}K_{\lambda \mu }(x^{-2})$ where $\lambda$ and $\mu$ are the partitions of *n* corresponding to $\chi$ and $\phi$, respectively, and $K_{\lambda \mu }(x)$ is the ordinary one-variable Kostka polynomial.

#### Corollary 2.5

The bigraded Hilbert series of ${\text {HP}}_*^{DR}(S_\phi \cap \mathcal {N})$ is

$$\begin{aligned} h({\text {HP}}_*^{DR}(S_\phi \cap \mathcal {N});x,y) = y^{\dim \mathcal {O}_\phi } \sum _{\chi } x^{\dim S_{\chi ,\phi }} {\text {ih}}_{\chi ,\phi }(x^{-1}) K_{\mathfrak {g},\chi }(y^{-2}), \end{aligned}$$

where the sum is taken over all $\chi \in {\text {Irrep}}(W)$ such that $\overline{\mathcal {O}_\chi } \supseteq \mathcal {O}_\phi$.

In the case $\mathfrak {g}=\mathfrak {sl}_n$, we obtain (in slightly rewritten form) a special case of a statement proved modulo Proudfoot’s conjecture in [19, Proposition 6.1]. (We show in the next subsection that the relevant case of Proudfoot’s conjecture also follows from our result.) Let $X_{\lambda ,\mu } := S_{\chi ,\phi }$ where $\lambda$ is the partition of *n* corresponding to $\chi$ and $\mu$ is the partition of *n* corresponding to $\phi$. Then $X_{(n)\mu }=S_\phi \cap \mathcal {N}$.

#### Corollary 2.6

The bigraded Hilbert series of ${\text {HP}}_*^{DR}(X_{(n)\mu })$ equals $y^{2n_\mu } \sum _{\nu \ge \mu } K_{\nu \mu }(x^2)K_{\nu (1^n)}(y^{-2})$.

Here $n_\mu = \sum _i (i-1)\mu _i$ is the partition statistic of $\mu$, which equals $\frac{1}{2}\dim \mathcal {O}_\phi$, and $\le$ is the dominance ordering on partitions.

## Proudfoot’s conjecture on symplectic duality

In [18, 3.4], Proudfoot conjectured that, in the case that *X* and $X^!$ are symplectic dual cones in the sense of [3, 10.15] (with Poisson brackets of degree two), then ${\text {HP}}_0(\mathcal {O}(X))\cong {\text {IH}}^*(X^!)$ as graded vector spaces. We deduce this now in a special case. Let $\mathfrak {g}=\mathfrak {sl}_r$ and let $\sigma$ be the sign representation of the symmetric group $W=\mathfrak {S}_r$. Let $\chi$ be an irreducible representation of *W* given by some partition $\lambda$ of *n*. Here and following we denote the dual partition of $\lambda$ by $\lambda ^t$. Let $\phi$ and $\phi '$ be nilpotent elements whose Jordan blocks are given by the parts of $\lambda$ and $\lambda ^t$, respectively. Let $X=S_\phi \cap \mathcal {N}$ and $X^! = \overline{\mathcal {O}_{\phi '}}$. The varieties *X* and $X^!$ are symplectically dual (cf., e.g., [3, §10.2.2]).

### Corollary 3.1

In the case above, ${\text {HP}}_0(\mathcal {O}(X))\cong {\text {IH}}^*(X^!)$.

### Proof

This follows from Corollary 2.1 by the proof of [19, Proposition 8.9]. Here is an outline for the reader’s convenience. By Corollary 2.1, $h({\text {HP}}_0(\mathcal {O}(X));y) = y^{\dim \mathcal {O}_\chi } K_{\mathfrak {g},\chi }(y^{-2})$. On the other hand, it is well known that $h({\text {IH}}^*(X^!);x) = x^{\dim \mathcal {O}_{\chi \otimes \sigma }}K_{\mathfrak {g},\chi \otimes \sigma }(x^{-2})$ (see (4.6), noting that in this case all of the local systems $L_\chi$ are trivial).

We thus need to establish the identity $t^{\dim \mathcal {O}_\chi }K_{\mathfrak {g},\chi }(t^{-2})=t^{\dim \mathcal {O}_{\chi \otimes \sigma }}K_{\mathfrak {g},\chi \otimes \sigma }(t^{-2})$, which is [19, (8.3)]. (It follows from palindromicity of $K_{\mathfrak {g},\chi }(t)$, Poincaré duality for $H^*(\mathcal {B})$, and the dimension formula for $\mathcal {O}_{\mathfrak {g},\chi }.)$

### Remark 3.2

By Corollary 2.2, we can also write a formula similar to the one above which holds for arbitrary type: as graded $\pi _0 {\text {Stab}}_G(\phi ) = \pi _1 \mathcal {O}_\phi$ representations,

$$\begin{aligned} {\text {HP}}_0(\mathcal {O}(S_\phi \cap \mathcal {N})) \cong \bigoplus _{\chi \in {\text {Irrep}}(W) \mid \mathcal {O}_\chi =\mathcal {O}_\phi } L_\chi \otimes {\text {IH}}^*(\mathcal {O}_\chi ,L_\chi ). \end{aligned}$$

## $\mathcal {D}$-module formulation and proof

### Recollections on $\mathcal {D}$-modules on $\mathcal {N}$

We consider two weakly $\mathbf {C}^\times$-equivariant $\mathcal {D}$-modules on $\mathcal {N}$, studied in [12]. The first is the pushforward, $\rho _* \varOmega _{T^*\mathcal {B}}$, which is actually strongly equivariant (since $\rho$ is equivariant). In [12], it is explained that this $\mathcal {D}$-module has a canonical *W*-action, and we obtain a decomposition of *W*-equivariant $\mathcal {D}$-modules,

$$\begin{aligned} \rho _* \varOmega _{T^*\mathcal {B}} \cong \bigoplus _{\chi \in {\text {Irrep}}(W)} \chi \otimes \mathrm {IC}(\mathcal {O}_\chi ,L_\chi ), \end{aligned}$$

where $(\mathcal {O}_\chi , L_\chi )$ is the pair of a nilpotent coadjoint orbit $\mathcal {O}_\chi$ and irreducible local system $L_\chi$ on $\mathcal {O}_\chi$; this is one way to define the Springer correspondence $\chi \mapsto (\mathcal {O}_\chi , L_\chi )$.

The second weakly $\mathbf {C}^\times$-equivariant $\mathcal {D}$-module on $\mathcal {N}$ is defined using the embedding $\mathcal {N}\subseteq \mathfrak {g}^*$. By definition (following Kashiwara), $\mathcal {D}$-modules on $\mathcal {N}$ are canonically identified with right $\mathcal {D}$-modules on $\mathfrak {g}^*$ supported on $\mathcal {N}$, via the maps $M \mapsto i_* M$ and $N \mapsto i^! N$. Since $\mathfrak {g}^*$ is smooth and affine, right $\mathcal {D}$-modules there can be defined as right modules over the ring $\mathcal {D}(\mathfrak {g}^*)$ of differential operators with polynomial coefficients. We have the map ${\text {ad}}: \mathfrak {g} \rightarrow \mathcal {D}(\mathfrak {g}^*)$, such that ${\text {ad}}(x)$ is the vector field acting by the adjoint action: precisely, ${\text {ad}}(x)(v)=[x,v]$ for $v \in \mathfrak {g} \subseteq {\text {Sym}}\,\mathfrak {g}= \mathcal {O}(\mathfrak {g}^*)$, which extends uniquely to a derivation.

Then, we consider the $\mathcal {D}$-module $M(\mathcal {N}) := i^!\bigl ( ({\text {ad}}(\mathfrak {g})+I(\mathcal {N}))\cdot \mathcal {D}(\mathfrak {g}^*) {\setminus } \mathcal {D}(\mathfrak {g}^*)\bigr )$. This is weakly $\mathbf {C}^\times$-equivariant with respect to the square of the dilation action on $\mathfrak {g}^* \supseteq \mathcal {N}$, since ${\text {ad}}(\mathfrak {g})$ and $I(\mathcal {N})$ are spanned by homogeneous elements. It is *not* strongly equivariant in general, since the Euler vector field need not be contained in $\mathcal {D}(\mathfrak {g}^*) \cdot ({\text {ad}}(\mathfrak {g}) + I(\mathcal {N}))$. (In fact, one can see that it is never strongly equivariant, for example using our main result below.)

#### Remark 4.1

The $\mathcal {D}$-module $M(\mathcal {N})$, called $\mathcal {M}_0$ in [12, § 6], can be identified with the $\mathcal {D}$-module $M(\mathcal {N})$ defined in [5] using the Poisson bracket on $\mathcal {O}(\mathcal {N})$. For the convenience of the reader, we recall the definition of the latter and the reason why it is the same as the $\mathcal {D}$-module we define (although we will not actually need it for the arguments of this paper); for a more detailed treatment see [4, 5]. For a general affine Poisson variety *X*, i.e., the spectrum of a Poisson algebra $\mathcal {O}(X)$, define $M(X) := H(X) \cdot \mathcal {D}_X {\setminus } \mathcal {D}_X$, where *H*(*X*) is the Lie algebra of Hamiltonian vector fields on *X*, and $\mathcal {D}_X$ is the standard $\mathcal {D}$-module on *X*. The latter can be defined for any embedding $i: X \rightarrow V$ into a smooth variety *V* as $\mathcal {D}_X = i^! (I_X \cdot \mathcal {D}(V) {\setminus } \mathcal {D}(V))$, with $\mathcal {D}(V)$ the ring of differential operators on *V* (viewed as a right module over itself) and $I_X \subseteq \mathcal {O}(X)$ the ideal of *X* (Kashiwara’s theorem implies this definition does not depend on the choice of *V*). The $\mathcal {D}$-module $\mathcal {D}_X$ has the property ${\text {Hom}}(\mathcal {D}_X, N) = \Gamma _{\mathcal {D}}(X,N)$, where $\Gamma _{\mathcal {D}}(X,N) := \{f \in \Gamma (V, i_* N) \mid I_X \cdot f = 0\}$ is the functor of sections scheme theoretically supported on *X*, which is independent of the choice of embedding as a consequence of the proof of Kashiwara’s theorem. This property could also be used to define $\mathcal {D}_X$. One then checks that *H*(*X*) acts as a Lie algebra on $\mathcal {D}_X$ via its left multiplication on $\mathcal {D}(V)$, which makes the definition given of *M*(*X*) sensible. Explicitly, $M(X) = i^!\bigl ( \mathcal {D}(V) \cdot (I_X + \widetilde{H(X)}) {\setminus } \mathcal {D}(V)\bigr )$, with $\widetilde{H(X)}$ the space of vector fields on *V* which are parallel to *X* and restrict there to elements of *H*(*X*). The $\mathcal {D}$-module *M*(*X*) has the defining property that ${\text {Hom}}(M(X),N) = \Gamma _{\mathcal {D}}(X,N)^{H(X)}$ for every $\mathcal {D}$-module *N* on *X*.

In the case $X=\mathcal {N}$ and $V=\mathfrak {g}^*$, actually *V* itself is Poisson. To identify $M(\mathcal {N})$ as in [5] with $({\text {ad}}(\mathfrak {g}) + I(\mathcal {N})) \cdot \mathcal {D}(\mathfrak {g}^*) {\setminus } \mathcal {D}(\mathfrak {g}^*)$, one can argue as follows. In this case, $\mathcal {N}\rightarrow \mathfrak {g}^*$ is a Poisson embedding, i.e., the Poisson bracket on $\mathfrak {g}^*$ preserves the ideal of $\mathcal {N}$ and induces the bracket on $\mathcal {N}$. In particular, $H(\mathcal {N})=H(\mathfrak {g}^*)|_{\mathcal {N}}$. Thus, it suffices to show that $({\text {ad}}(\mathfrak {g}) + I(\mathcal {N})) \cdot \mathcal {D}(\mathfrak {g}^*) = (H(\mathfrak {g}^*)+I(\mathcal {N})) \cdot \mathcal {D}(\mathfrak {g}^*)$. In fact, more is true: ${\text {ad}}(\mathfrak {g}) \cdot \mathcal {D}(\mathfrak {g}^*) = H(\mathfrak {g}^*) \cdot \mathcal {D}(\mathfrak {g}^*)$, and this holds for an arbitrary finite-dimensional Lie algebra $\mathfrak {g}$. To see this, first observe that ${\text {ad}}(\mathfrak {g}) \subseteq H(\mathfrak {g}^*)$, so we only need to show that $H(\mathfrak {g}^*) \subseteq {\text {ad}}(\mathfrak {g}) \cdot \mathcal {D}(\mathfrak {g}^*)$. Next, for every $f \in \mathcal {O}(\mathfrak {g}^*)$, we have $\xi _f = \sum _i f_i {\text {ad}}(x_i)$ for some $f_i \in \mathcal {O}(\mathfrak {g}^*)$ and $x_i \in \mathfrak {g}$. Then, in $\mathcal {D}(\mathfrak {g}^*)$, we have $\xi _f = \sum _i {\text {ad}}(x_i) \cdot f_i - \sum _i \{x_i,f_i\}$. Finally, one observes that $\sum _i \{x_i, f_i\} = 0$ since $\xi _f$ was Hamiltonian. Thus, $H(\mathfrak {g}^*) \subseteq {\text {ad}}(\mathfrak {g}) \cdot \mathcal {D}(\mathfrak {g}^*)$, as desired.

### Hotta and Kashiwara’s theorem

We recall the following result of Hotta and Kashiwara (the case $\lambda =0$ of  [12, Theorem 6.1]). Let $\mathrm {Har}(\mathfrak {h}^*) \subseteq \mathcal {O}(\mathfrak {h}^*)={\text {Sym}}\,\mathfrak {h}$ be the subspace of harmonic polynomials, i.e.,

$$\begin{aligned} \mathrm {Har}(\mathfrak {h}^*) := \{f \in \mathcal {O}(\mathfrak {h}^*) \mid P(\partial _x)(f) = 0, \forall P \in ({\text {Sym}}\,\mathfrak {h}^*)^W_+\}, \end{aligned}$$

where we denote the embedding ${\text {Sym}}\,\mathfrak {h}^* \hookrightarrow \mathcal {D}(\mathfrak {h}^*)$ as constant coefficient differential operators by $P \mapsto P(\partial _x)$, $({\text {Sym}}\,\mathfrak {h}^*)^W$ is the *W*-invariant subalgebra, and $({\text {Sym}}\,\mathfrak {h}^*)^W_+$ is the augmentation ideal (of operators whose constant term is zero).

Let $\sigma$ be the sign representation of *W*, placed in degree zero.

#### Theorem 4.2

(Hotta and Kashiwara) There is a canonical isomorphism of weakly equivariant $\mathcal {D}$-modules,

4.1 $$\begin{aligned} M(\mathcal {N}) \mathop {\longrightarrow }\limits ^{\sim } {\text {Hom}}_W \left( \mathrm {Har}(\mathfrak {h}^*) \otimes \sigma , \rho _* \varOmega _{T^*\mathcal {B}} \right) . \end{aligned}$$

The above result follows from Theorem 5.2, Proposition 6.3.1, and Theorem 6.1 of [12], and the canonical isomorphism is originally constructed as

$$\begin{aligned} M(\mathcal {N})^F \mathop {\longrightarrow }\limits ^{\sim } {\text {Hom}}_W \left( \mathrm {Har}(\mathfrak {h}), (\rho _* \varOmega _{T^*\mathcal {B}})^F \otimes \sigma \right) \end{aligned}$$

where the superscript *F* denotes the Fourier transform. (There also the $\mathcal {D}$-modules are on $\mathfrak {g}$ and $\mathfrak {h}$ rather than $\mathfrak {g}^*$ and $\mathfrak {h}^*$, so $\mathrm {Har}(\mathfrak {h}^*)$ appears.) But, for a weakly $\mathbb {C}^{\times }$-equivariant $\mathcal {D}$-module *M* and graded vector space *V*, the fact that $F({\text {Eu}}_{\mathfrak {g}^*}) = - {\text {Eu}}_{\mathfrak {g}^*} - \dim \mathfrak {g}$ implies that $(M \otimes V)^F \cong M^F \otimes V^*$. We recover the statement of the theorem.

### Proof of Theorem 1.1

Theorem 1.1 is an immediate application of Hotta and Kashiwara’s theorem. Namely, pushing forward to a point, $H^{-i} \pi _* M(\mathcal {N}) = {\text {HP}}_i^{DR}(\mathcal {N})$ and

$$\begin{aligned} H^{-i} \pi _* {\text {Hom}}_W \left( \mathrm {Har}(\mathfrak {h}^*) \otimes \sigma , \rho _* \varOmega _{T^*\mathcal {B}} \right)= & {} {\text {Hom}}_W({\text {Sym}}\,\mathfrak {h}/(({\text {Sym}}\,\mathfrak {h})_+^W) \otimes \sigma ,\\&\times H^{2 \dim \mathcal {B}- i} (T^*\mathcal {B})). \end{aligned}$$

### Proof of Theorem 1.3

To prove Theorem 1.3, we construct a $\mathbf {C}^\times$-equivariant vector bundle on $\mathfrak {h}^*/W$. Let $\chi : \mathfrak {g}^* \rightarrow \mathfrak {g}^*//G \displaystyle \mathop {\rightarrow }^\sim \mathfrak {h}^*/W$ be the coadjoint quotient composed with the Chevalley isomorphism. Consider the $(\mathcal {O}(\mathfrak {g}^*)^G, \mathcal {D}(\mathfrak {g}^*))$-bimodule $\widetilde{M} := {\text {ad}}(\mathfrak {g}) \mathcal {D}(\mathfrak {g}^*) {\setminus } \mathcal {D}(\mathfrak {g}^*)$, equipped with the $\mathcal {O}(\mathfrak {g}^*)^G$-structure by left multiplication by *G*-invariant functions. This has the property that, for every $\bar{\lambda } \in \mathfrak {g}^*//G$, the fiber $\bar{\lambda } \otimes _{\mathcal {O}(\mathfrak {g}^*)^G} \widetilde{M}$ is nothing but $M(\chi ^{-1}(\bar{\lambda }))$. Moreover, by [14, Theorem 1.2], $\widetilde{M}$ is flat over $\mathcal {O}(\mathfrak {g}^*)^G$. (Note that the theorem there actually applies to the corresponding left $\mathcal {D}(\mathfrak {g}^*)$-module $\mathcal {D}(\mathfrak {g}^*) / \mathcal {D}(\mathfrak {g}^*) {\text {ad}}(\mathfrak {g}) = \widetilde{M} \otimes _{\mathcal {O}_{\mathfrak {g}^*}} \varOmega _{\mathfrak {g}^*}$, but its flatness is equivalent to that of $\widetilde{M}$.) Hence, this tensor product is derived: $\bar{\lambda } \otimes _{\mathcal {O}(\mathfrak {g})^G} \widetilde{M} = H^0(\bar{\lambda } \otimes ^{L}_{\mathcal {O}(\mathfrak {g})^G} \widetilde{M})$.

We can now take the $\mathcal {D}$-module pushforward to a point (under $\pi : \mathfrak {g}\rightarrow {\mathsf {Spec}}\mathbf {C}$), to obtain $\pi _* \widetilde{M}$, a quasi-coherent sheaf on $\mathfrak {g}^*//G \cong \mathfrak {h}^*/W$ whose fibers are precisely the Poisson-de Rham homologies:

$$\begin{aligned} H^{-i} (\bar{\lambda } \otimes ^L_{\mathcal {O}(\mathfrak {g})^G} \pi _* \widetilde{M}) \cong H^{-i} (\pi _* (\bar{\lambda } \otimes ^L_{\mathcal {O}(\mathfrak {g})^G} \widetilde{M})) \cong H^{-i} (\pi _* \widetilde{M}|_{\bar{\lambda }}) = {\text {HP}}_{i}^{DR}(\chi ^{-1}(\bar{\lambda })). \end{aligned}$$

Here, the first isomorphism follows from associativity of derived tensor product, since $\pi _*(N) = N \otimes _{\mathcal {D}(\mathfrak {g}^*)} \mathcal {O}(\mathfrak {g}^*)$ for every right $\mathcal {D}(\mathfrak {g}^*)$-module *N*. For $\lambda \in \mathfrak {h}_{{\text {reg}}}^*$, the variety $\chi ^{-1}(\bar{\lambda })$ is symplectic and hence ${\text {HP}}_i^{DR}(\chi ^{-1}(\bar{\lambda })) = H^{\dim \chi ^{-1}(\bar{\lambda }) - i}(\chi ^{-1}(\bar{\lambda }))$ (by [5, Example 2.6, Remark 2.14]).

Next, consider the Grothendieck–Springer simultaneous resolution $\widetilde{\mathfrak {g}}^* \rightarrow \mathfrak {g}^*$. Let $\mathfrak {n} := [\mathfrak {b}, \mathfrak {b}]$. Recall that $\widetilde{\mathfrak {g}}^* := \{(\mathfrak {b},\phi ) \in \mathcal {B} \times \mathfrak {g}^* \mid \phi \in \mathfrak {n}^\perp \}$. Let ${\text {pr}}_1: \widetilde{\mathfrak {g}}^* \rightarrow \mathcal {B}$ and ${\text {pr}}_2: \widetilde{\mathfrak {g}}^* \rightarrow \mathfrak {g}^*$ be the two projections. There is also a canonical map $\widetilde{\pi }: \widetilde{\mathfrak {g}}^* \rightarrow \mathfrak {h}^*$, given by $\widetilde{\pi }(\mathfrak {b},\phi ) = \phi |_{\mathfrak {b}/\mathfrak {n}}$. We can consider the composition $\widetilde{\mathfrak {g}}^* \rightarrow \mathcal {B} \times \mathfrak {h}^* \rightarrow \mathfrak {h}^*$, with the first map $({\text {pr}}_1 \times \widetilde{\pi })$. The first map makes $\widetilde{\mathfrak {g}}^*$ a principal $\mathfrak {n}$-bundle over $\mathcal {B} \times \mathfrak {h}^*$. The second map is the projection. Put together, we obtain a canonical isomorphism $H^*(\widetilde{\pi }^{-1}(\lambda )) \cong H^*(\mathcal {B})$ for all $\lambda$. Furthermore, when $\lambda \in \mathfrak {h}_{{\text {reg}}}^*$, ${\text {pr}}_2$ induces an isomorphism $\displaystyle \widetilde{\pi }^{-1}(\lambda ) \displaystyle \mathop {\rightarrow }^\sim \chi ^{-1}(\bar{\lambda }) \subseteq \mathfrak {g}^*$. Therefore, for regular $\lambda$ we obtain canonical isomorphisms $H^{*}(\chi ^{-1}(\bar{\lambda })) \cong H^*(\mathcal {B})$. (Note that these depend on $\lambda \in \mathfrak {h}_{{\text {reg}}}^*$, and not just on the *W*-orbit $\bar{\lambda }$.)

Let $q: \mathfrak {h}^* \rightarrow \mathfrak {h}^*/W$ be the quotient. We can consider the pullback $q^* \pi _* \widetilde{M} = \mathcal {O}(\mathfrak {h}^*) \otimes _{\mathcal {O}(\mathfrak {h}^*)^W} \pi _* \widetilde{M}$. The previous paragraphs show that $H^{-i}(q^* \pi _* \widetilde{M})|_{\mathfrak {h}_{{\text {reg}}}^*}$ canonically identifies with the Gauss–Manin system $H^{2\dim \mathcal {B}- i}(\widetilde{\pi }^{-1}(\lambda ))$ over $\mathfrak {h}_{{\text {reg}}}^*$, and that every fiber is canonically identified with $H^{2 \dim \mathcal {B}-i}(\mathcal {B})$.

#### Lemma 4.3

The sheaf $\pi _* \widetilde{M}$ is a finite rank $\mathbf {C}^\times$-equivariant vector bundle on $\mathfrak {h}^* / W$.

#### Proof

First note that, by [14, Theorem 1.1], $\widetilde{M}$ is a $\mathcal {D}(\mathfrak {h}^*)^W$-module. Let $N := \varOmega _{\mathfrak {h}^*} \otimes _{\mathcal {O}(\mathfrak {h}^*)} \mathcal {D}(\mathfrak {h}^*) \otimes _{\mathcal {D}(\mathfrak {h}^*)^W} \widetilde{M}$, a right $\mathcal {D}(\mathfrak {h}^* \times \mathfrak {g}^*)$-module. We can furthermore consider an alternative description of *N* from [12]: Let $\iota _1: \mathcal {O}(\mathfrak {h}^*)^W \rightarrow \mathcal {O}(\mathfrak {g}^*)^G$ and $\iota _2: ({\text {Sym}}\,\mathfrak {h}^*)^W \rightarrow ({\text {Sym}}\,\mathfrak {g}^*)^G$ be the Chevalley isomorphisms (considering ${\text {Sym}}\,\mathfrak {h}^* \subseteq \mathcal {D}(\mathfrak {h}^*)$ the constant coefficient operators and similarly for $\mathfrak {g}^*$). Define

$$\begin{aligned} N'= & {} ({\text {ad}}(\mathfrak {g}) \mathcal {D}(\mathfrak {h}^* \times \mathfrak {g}^*) + \{P - \iota _1(P) \mid P \in \mathcal {O}(\mathfrak {h}^*)^W\} \mathcal {D}(\mathfrak {h}^* \times \mathfrak {g}^*) \\&+ \{Q - \iota _2(Q) \mid Q \in ({\text {Sym}}\,\mathfrak {h}^*)^W \}\mathcal {D}(\mathfrak {h}^* \times \mathfrak {g}^*)) {\setminus } \mathcal {D}(\mathfrak {h}^* \times \mathfrak {g}^*). \end{aligned}$$

This is the right corresponding to the left $\mathcal {D}(\mathfrak {g}^* \times \mathfrak {h}^*)$-module denoted $\mathcal {N}$ in [12, Theorem 4.2]. By [12, Theorem 4.2], we know that $N'$ is a simple holonomic $\mathcal {D}(\mathfrak {h}^* \times \mathfrak {g}^*)$-module, and moreover,

4.2 $$\begin{aligned} N' \cong f_* \varOmega _{\widetilde{\mathfrak {g}}^*} \end{aligned}$$

where $f = \widetilde{\pi } \times {\text {pr}}_2: \widetilde{\mathfrak {g}^*} \rightarrow \mathfrak {h}^* \times \mathfrak {g}^*$. By definition, there is a surjection $N' \rightarrow N$, and since $N'$ is simple, $N \cong N'$. Now letting $\pi ': \mathfrak {h}^* \times \mathfrak {g}^* \rightarrow \mathfrak {h}^*$ be the first projection, we have $\pi '_* N \cong \widetilde{\pi }_* \varOmega _{\widetilde{\mathfrak {g}^*}}$. Decomposing $\widetilde{\pi }$ as the composition $\widetilde{\mathfrak {g}^*} \rightarrow \mathcal {B}\times \mathfrak {h}^* \rightarrow \mathfrak {h}^*$, we obtain that the right $\mathcal {D}$-module $H^{-i}\pi '_* N$ corresponds (via the right–left $\mathcal {D}$-module correspondence followed by the Riemann–Hilbert correspondence) to the trivial local system on $\mathfrak {h}^*$ with fibers $H^{2\dim \mathcal {B}-i}(\mathcal {B})$.

Finally, by definition, we have

4.3 $$\begin{aligned} \pi '_* N = \varOmega _{\mathfrak {h}^*} \otimes _{\mathcal {O}(\mathfrak {h}^*)} \mathcal {D}(\mathfrak {h}^*) \otimes _{\mathcal {D}(\mathfrak {h}^*)^W} \pi _* \widetilde{M}. \end{aligned}$$

It is well known that $\mathcal {D}(\mathfrak {h}^*)$ is an invertible $(\mathcal {D}(\mathfrak {h}^*)^W, \mathcal {D}(\mathfrak {h}^*) \rtimes W)$-bimodule, inducing a Morita equivalence between the two algebras. In particular, $\mathcal {D}(\mathfrak {h}^*)$ is a projective $\mathcal {D}(\mathfrak {h}^*)^W$-module. Thus, $\pi _* \widetilde{M}$ is itself a $\mathcal {D}(\mathfrak {h}^*)^W$-module summand of $\pi '_* N$. The latter is a graded finitely generated projective $\mathcal {O}(\mathfrak {h}^*)$-module and hence also a finitely generated projective $\mathcal {O}(\mathfrak {h}^*)^W$-module. Thus, so is $\pi _* \widetilde{M}$. In other words, $\pi _* \widetilde{M}$ is a $\mathbf {C}^\times$-equivariant finite rank vector bundle over $\mathfrak {h}^*/W$. This completes the proof of the lemma.

Observe that, since the map *q* is flat and $\mathbf {C}^\times$-equivariant, Lemma 4.3 implies that $q^* \pi _* \widetilde{M}$ is a finite rank $\mathbf {C}^\times$-equivariant vector bundle on $\mathfrak {h}^*$. For every $\lambda \in \mathfrak {h}^*_{{\text {reg}}}$, we can restrict $q^* \pi _* \widetilde{M}$ to the line $\mathbf {C}\cdot \lambda$, and we obtain a $\mathbf {C}^\times$-equivariant vector bundle on the line $\mathbf {A}^1$, $L_\lambda := (q^* \pi _* \widetilde{M})|_{\mathbf {C}\cdot \lambda }$. But finite rank $\mathbf {C}^\times$-equivariant vector bundles on the line are well known to be the same thing as finite-dimensional filtered vector spaces: given a finite-dimensional filtered vector space $F^\cdot V$, one takes the associated $\mathbf {C}[t]$-module $L = \bigoplus _{i \in \mathbf {Z}} F^{\le i} V \cdot t^i$, and the opposite direction is given by setting $V := L|_{t=1}$ and taking the filtration by the image of weight spaces $\bigoplus _{j \le i} L_j$. Moreover, the fiber $L|_0$ at zero is the associated graded vector space ${\text {gr}}V$. Therefore, $(q^* \pi _* \widetilde{M})|_{\mathbf {C}\cdot \lambda }$ is nothing but a collection of filtrations on the underlying cohomologies $H^{-i}(q^* \pi _* \widetilde{M})|_{\lambda } \cong H^{2\dim \mathcal {B}-i}(\mathcal {B})$. This produces the desired filtrations on the cohomology of the flag variety. The associated graded vector spaces are $H^{-i} (q^* \pi _* \widetilde{M})|_{0} = {\text {HP}}^{DR}_i(\mathcal {N})$.

It remains to check the *W*-equivariance. First, $(q^* \pi _* \widetilde{M})|_{\lambda } = (q^* \pi _* \widetilde{M})_{w(\lambda )}$ for all $\lambda$. On the other hand, to obtain a filtration on the flag variety cohomology, we identify $(q^* \pi _* \widetilde{M})|_{\mathfrak {h}_{{\text {reg}}}^*}$ with the Gauss–Manin system of $\widetilde{\pi }^{-1}(\mathfrak {h}_{{\text {reg}}}^*) \rightarrow \mathfrak {h}_{{\text {reg}}}^*$. The latter is *W*-equivariant, and the composition $H^*(\widetilde{\pi }^{-1}(\lambda )) \cong H^*(\mathcal {B}) \cong H^*(\widetilde{\pi }^{-1}(w(\lambda ))) \cong H^*(\widetilde{\pi }^{-1}(\lambda ))$, of applying the Gauss–Manin connection twice followed by the isomorphism $\widetilde{\pi }^{-1}(w(\lambda )) \cong \chi ^{-1}(\bar{\lambda }) \cong \widetilde{\pi }^{-1}(\lambda )$, is the action of *w*.

### Proof of Corollary 1.4

The proof consists of studying the relationship between the right $\mathcal {D}(\mathfrak {h}^*)$-module $\pi '_*N$ and the left $\mathcal {D}(\mathfrak {h}^*)^W$-module $\pi _* \widetilde{M}$. Applying the Morita equivalence between $\mathcal {D}(\mathfrak {h}^*) \rtimes W$ and $\mathcal {D}(\mathfrak {h}^*)^W$ defined by $\mathcal {D}(\mathfrak {h}^*)$ to the expression (4.3) for $\pi '_* N$ implies that

$$\begin{aligned} \pi _* \widetilde{M} = \mathcal {D}(\mathfrak {h}^*) \otimes _{\mathcal {D}(\mathfrak {h}^*) \rtimes W} \varOmega _{\mathfrak {h}^*}^{-1} \otimes _{\mathcal {O}(\mathfrak {h}^*)} \pi '_* N = (\varOmega _{\mathfrak {h}^*}^{-1} \otimes _{\mathcal {O}(\mathfrak {h}^*)} \pi '_* N)^W. \end{aligned}$$

More generally, we can consider the functor from weakly $\mathbf {C}^\times \times W$-equivariant right $\mathcal {D}(\mathfrak {h}^*)$-modules to (weakly) $\mathbf {C}^\times$-equivariant left $\mathcal {D}(\mathfrak {h}^*)^W$-modules:

$$\begin{aligned} T: \mathcal {F} \mapsto (\varOmega _{\mathfrak {h}^*}^{-1} \otimes _{\mathcal {O}(\mathfrak {h}^*)} \mathcal {F})^W. \end{aligned}$$

For every irreducible representation $\chi$ of *W*, we can form the strongly $\mathbf {C}^\times \times W$-equivariant right $\mathcal {D}(\mathfrak {h}^*)$-module, $\mathcal {F}_\chi := \varOmega _{\mathfrak {h}^*} \otimes \chi$ (which under the Riemann–Hilbert correspondence is the trivial local system $\chi$ on $\mathfrak {h}^*$ equipped with the *W*-linearization given by the representation). We obtain the formula:

$$\begin{aligned} T(\mathcal {F}_\chi ) = (\chi \otimes \mathcal {O}(\mathfrak {h}^*))^W. \end{aligned}$$

This is a $\mathbf {C}^\times$-equivariant vector bundle on $\mathfrak {h}^*/W$. For any $\lambda \in \mathfrak {h}^*_{{\text {reg}}}$, we can restrict $T(\mathcal {F}_\chi )$ to the line $\mathbf {C}\cdot \bar{\lambda }$ and get a $\mathbf {C}^\times$-equivariant vector bundle on the line, i.e., a finite-dimensional filtered vector space $T(\mathcal {F}_\chi )|_{\bar{\lambda }}$. There is a canonical isomorphism of vector spaces

$$\begin{aligned} T(\mathcal {F}_\chi )|_{\bar{\lambda }} \displaystyle \mathop {\rightarrow }^\sim \mathcal {F}_\chi |_{\lambda }, \end{aligned}$$

obtained from the composition

$$\begin{aligned} (\varOmega _{\mathfrak {h}^*}^{-1} \otimes _{\mathcal {O}(\mathfrak {h}^*)} \mathcal {F}_\chi )^W \hookrightarrow \varOmega _{\mathfrak {h}^*}^{-1} \otimes _{\mathcal {O}(\mathfrak {h}^*)} \mathcal {F}_\chi \mathop {\rightarrow }^{\text {vol}_{\mathfrak {h}^*} \otimes -} \mathcal {F}, \end{aligned}$$

by applying the restriction of the source to $\bar{\lambda }$ and the target to $\lambda$. This is an isomorphism because, for every *W*-equivariant vector bundle $\mathcal {F}$ on $\mathfrak {h}^*$ and every $\lambda \in \mathfrak {h}_{{\text {reg}}}^*$, the fiber of $\mathcal {F}^W$ at $\bar{\lambda }$ equals the fiber of $\mathcal {F}$ at $\lambda$.

Put together, for every $\lambda \in \mathfrak {h}^*_{{\text {reg}}}$, we obtain a canonical filtration on the fiber $\mathcal {F}_\chi |_{\lambda }$.

Next, note that every weakly $\mathbf {C}^\times \times W$-equivariant $\mathcal {O}_{\mathfrak {h}^*}$-coherent right $\mathcal {D}_{\mathfrak {h}^*}$-module is a direct sum of shifts $\mathcal {F}_\chi (k)$, where the notation indicates a grading shift: $M(k) := M \otimes _\mathbf {C}\mathbf {C}_{-k}$, where $k \in \mathbf {Z}$ and $\mathbf {C}_k$ is the representation of $\mathbf {C}^\times$ in which $\gamma \in \mathbf {C}^\times$ acts by $\gamma ^k \cdot {\text {Id}}$. In the strongly equivariant case, $k=0$ for every summand, i.e., these modules are direct sums of copies of $\mathcal {F}_\chi$.

Returning to $\pi '_* N$, we can write $\pi '_* N$ as a direct sum of such $\mathcal {F}_\chi$ (with homological and weight shifts). Let us determine the weight shifts. Recall from (4.2) and the preceding that *N* is a simple holonomic right $\mathcal {D}_{\mathfrak {h}^* \times \mathfrak {g}^*}$-module isomorphic to $f_* \varOmega _{\widetilde{\mathfrak {g}}^*}$. Equip the latter with the canonical strong $\mathbf {C}^\times$-equivariant structure coming from this structure on $\varOmega _{\widetilde{\mathfrak {g}}^*}$. Then we can see from the proof of [12, Theorem 4.2] that the isomorphism $N \rightarrow f_*\varOmega _{\widetilde{\mathfrak {g}}^*}$ is *W*-equivariant and sends the generator $[1] \in N$ to an element of degree $2 \dim \mathcal {B}$. Thus, if we put $[1] \in N$ in degree zero, we obtain that $N \cong f_* \varOmega _{\widetilde{\mathfrak {g}}^*}(2 \dim \mathcal {B})$ as a weakly $\mathbf {C}^\times \times W$-equivariant $\mathcal {D}$-module. Alternatively (but which amounts to the same proof), we observe that, since *N* is simple, there is only a single value of the shift, and then it must be $2 \dim \mathcal {B}$ in order to agree with Theorem 1.1. Pushing forward, all weight shifts of the $\mathcal {F}_\chi$ appearing in $\pi '_* N$ are by $2 \dim \mathcal {B}$.

It follows that the filtration on each cohomology of the fiber at $\lambda$, $H^{-i}(\pi '_* N|_{\lambda }) = H^{2\dim \mathcal {B}-i}(\mathcal {B})$, is a direct sum of copies of a single filtered vector space for each irreducible representation $\chi$ of *W*, and the associated graded vector space ${\text {gr}}\mathcal {F}_\chi |_{\lambda }$ of the latter is isomorphic to $(\chi \otimes \mathcal {O}(\mathfrak {h}^*))^W|_{0} \otimes \mathbf {C}_{-2\dim \mathcal {B}}$. Recall that, by Chevalley’s theorem, $\mathcal {O}(\mathfrak {h}^*)$ is a free $\mathcal {O}(\mathfrak {h}^*)^W$-module, and in fact $\mathcal {O}(\mathfrak {h}^*) \cong (\mathcal {O}(\mathfrak {h}^*) / (\mathcal {O}(\mathfrak {h}^*)^W)_+) \otimes \mathcal {O}(\mathfrak {h}^*)^W$ as a $\mathcal {O}(\mathfrak {h}^*)^W \times W$-module. Put together, the Hilbert series of ${\text {gr}}\mathcal {F}_\chi |_{\lambda }$ is

4.4 $$\begin{aligned} h((\chi \otimes \mathcal {O}_{\mathfrak {h}^*}(2\dim \mathcal {B}))^W|_{0}; y)= & {} y^{-2\dim \mathcal {B}} h({\text {Hom}}_W(\chi , \mathcal {O}_{\mathfrak {h}^*}/((\mathcal {O}_{\mathfrak {h}^*}^W)_+)); y) \nonumber \\= & {} h({\text {Hom}}_W(\chi \otimes \sigma , \mathcal {O}_{\mathfrak {h}^*}/((\mathcal {O}_{\mathfrak {h}^*}^W)_+)); y^{-1})\nonumber \\= & {} K_{\mathfrak {g},\chi }(y^{-2}), \end{aligned}$$

where the second equality is due to Poincaré duality for $H^*(\mathcal {B}) \cong \mathcal {O}_{\mathfrak {h}^*}/((\mathcal {O}_{\mathfrak {h}^*}^W)_+)$.

### The structure of $M(\mathcal {N})$

The following statement was conjectured in [19, Conjecture 8.1]. As in Sect. 2, let $V_\chi ^*$ be a weight-graded vector space with Hilbert series $K_{\mathfrak {g},\chi }(y^{-2})$. Since $\rho _* \varOmega _{T^*\mathcal {B}}$ is strongly $\mathbf {C}^\times$-equivariant and ${\text {IC}}(\mathcal {O}_\chi ,L_\chi )$ is a summand for all $\chi \in {\text {Irrep}}(W)$, it follows that ${\text {IC}}(\mathcal {O}_\chi ,L_\chi )$ admits the structure of a strongly $\mathbf {C}^\times$-equivariant $\mathcal {D}$-module. Let us equip it with this structure.

#### Theorem 4.4

There is an isomorphism of weakly equivariant $\mathcal {D}$-modules,

$$\begin{aligned} M(\mathcal {N}) \cong \bigoplus _{\chi \in {\text {Irrep}}(W)} V_\chi ^* \otimes {\text {IC}}(\mathcal {O}_\chi ,L_\chi ). \end{aligned}$$

#### Proof

The proof follows from Hotta and Kashiwara’s Theorem 4.2. We need to observe that $\mathrm {Har}(\mathfrak {h}^*)$ is canonically isomorphic (as a graded *W*-representation) to ${\text {Sym}}\,\mathfrak {h}/ (({\text {Sym}}\,\mathfrak {h})_+^W)$ and thus to $H^*(\mathcal {B})$ as a graded *W*-representation. So we get

4.5 $$\begin{aligned}&{\text {Hom}}_W(\mathrm {Har}(\mathfrak {h}^*) \otimes \sigma , \rho _* \varOmega _{T^*\mathcal {B}}) \cong {\text {Hom}}_W(H^{*}(\mathcal {B}) \otimes \sigma , \rho _* \varOmega _{T^*\mathcal {B}}) \nonumber \\&\quad \cong \bigoplus _{\chi \in {\text {Irrep}}(W)} {\text {Hom}}_W(\chi \otimes \sigma , H^*(\mathcal {B}))^* \otimes {\text {Hom}}_W(\chi , \rho _* \varOmega _{T^*\mathcal {B}}). \end{aligned}$$

Then, by definition, ${\text {Hom}}_W(\chi , \rho _* \varOmega _{T^*\mathcal {B}}) \cong {\text {IC}}(\mathcal {O}_{\chi },L_{\chi })$. By Poincaré duality, ${\text {Hom}}_W(\chi \otimes \sigma , H^*(\mathcal {B})) \cong {\text {Hom}}_W(\chi , H^{2\dim \mathcal {B}-*}(\mathcal {B}))$, and the Hilbert series of the latter is $K_{\mathfrak {g},\chi }(t^2)$, so its dual has Hilbert series $K_{\mathfrak {g},\chi }(y^{-2})$. Thus, the RHS is isomorphic to $\bigoplus _\chi V_\chi ^* \otimes {\text {IC}}(\mathcal {O}_{\chi },L_\chi )$, as desired. $\square$

### Proof of Lusztig’s formula

Pushing $\rho _* \varOmega _{T^*\mathcal {B}} \cong \bigoplus _{\chi \in {\text {Irrep}}(W)} \chi \otimes {\text {IC}}(\mathcal {O}_{\chi },L_\chi )$ to a point, we get

4.6 $$\begin{aligned} x^{-\dim \mathcal {O}_\chi }h({\text {IH}}^{*}(\mathcal {O}_\chi ,L_\chi );x) = x^{-2\dim \mathcal {B}}h({\text {Hom}}_W(\chi ,H^{*}(T^*\mathcal {B}));x)= K_{\mathfrak {g},\chi }(x^{-2}). \end{aligned}$$

Thus, pushing the formula of Theorem 4.4 to a point, we obtain Lusztig’s formula (1.1).

## Proofs of results on *W*-algebras and Slodowy slices

### Proof of Corollaries 2.1 and 2.2

The first assertion of Corollary 2.1 follows by [19, Remark 8.7]. (In more detail, it is a consequence of Theorem 4.4 and [19, Theorem 5.1].) The assertion for $W_\phi ^\eta$ then follows immediately from [6, Theorem 1.10.(ii)], stating that ${\text {HP}}_0(W_\phi ^\eta )$ is flat in $\eta$. Hence, as explained in [6, Theorem 1.10.(iii)] and its proof, ${\text {HP}}_0(W_\phi )$ is a free graded module over $\mathcal {O}(\mathfrak {g})^\mathfrak {g}$, so the assertion follows for ${\text {HP}}_0(W_\phi )$ as well. The same argument implies the refined statement, Corollary 2.2.

### Proof of Corollary 2.5

For Corollary 2.5, we need to compute $M(S_\phi \cap \mathcal {N})$ as a weakly $\mathbf {C}^\times$-equivariant $\mathcal {D}$-module, with respect to its dilation action with fixed point $\phi$. We will use the notation *N*(*k*) for the shift of the weak $\mathbf {C}^\times$-equivariant structure on *N* as defined in Sect. 4.5.

#### Proposition 5.1

As weakly $\mathbf {C}^\times$-equivariant $\mathcal {D}$-modules,

$$\begin{aligned} M(S_\phi \cap \mathcal {N})(\dim \mathcal {O}_\phi ) \cong \bigoplus _\chi V_\chi ^* \otimes {\text {IC}}(S_{\chi ,\phi },L_\chi |_{S_{\phi } \cap \mathcal {O}_{\chi }}), \end{aligned}$$

the sum taken over $\chi \in {\text {Irrep}}(W)$ such that $\overline{\mathcal {O}_\chi } \supseteq \mathcal {O}_\phi$.

#### Proof

Completing at $\phi$, we have, by the Darboux–Weinstein decomposition theorem, as formal Poisson schemes, $\hat{\mathcal {N}} \cong \widehat{S_\phi \cap \mathcal {N}} \times \hat{\mathcal {O}}_{\phi }$. But $\mathcal {O}_{\phi }$ is smooth so $\hat{\mathcal {O}}_{\phi }$ is the completion of a symplectic vector space at the origin. So, disregarding the equivariant structure, $M(\mathcal {N})|_{\hat{\mathcal {N}}} \cong M(S_\phi \cap \mathcal {N})|_{\widehat{S_\phi \cap \mathcal {N}}} \boxtimes \varOmega _{\mathcal {O}_\phi }|_{\hat{\mathcal {O}}_{\phi }}$. As a result, $M(S_\phi \cap \mathcal {N})$ is a direct sum of intermediate extensions of local systems on its leaves, which are $S_\phi \cap \mathcal {O}_\chi$; the local systems which appear are $K_{S_\phi \cap \mathcal {O}_\chi } := H^0 i_{S_\phi \cap \mathcal {O}_\chi }^* M(S_\phi \cap \mathcal {N})$ where $i_{S_\phi \cap \mathcal {O}_\chi }: S_\phi \cap \mathcal {O}_\chi \rightarrow (S_\phi \cap \mathcal {N})$ is the inclusion. This is even true together with the equivariant structure. By [19, Theorem 5.1], the shift $K_{S_\phi \cap \mathcal {O}_\chi }(-\dim (S_\phi \cap \mathcal {O}_\chi ))$ is the weakly equivariant local system described in [5, § 4.3] canonically given by attaching, to each $x \in S_\phi \cap \mathcal {O}_\chi$, the fiber ${\text {HP}}_0(\mathcal {O}(S_x))$ where $S_x$ is the slice to $S_\phi \cap \mathcal {O}_\chi$ in $S_{\phi } \cap \mathcal {N}$ at *x*.

Precisely, the summand of $K_{S_\phi \cap \mathcal {O}_\chi }$ which is weakly equivariant with respect to the character $m-\dim (S_\phi \cap \mathcal {O}_\chi )$ of $\mathbf {C}^\times$ is the local system attaching to each *x* the weight *m* subspace of ${\text {HP}}_0(\mathcal {O}(S_x))$.

Note that $S_x$ is isomorphic to the same slice to $\mathcal {O}_{\chi }$ in $\mathcal {N}$ at *x*, so this is compatible with our previous notation. Passing back to $\mathcal {N}$, we know again that $M(\mathcal {N})$ is a direct sum of intermediate extensions of local systems $L_{\mathcal {O}_\psi }$ on its leaves $\mathcal {O}_\psi$. Again applying [19, Theorem 5.1], $K_{\mathcal {O}_\psi }(-\dim \mathcal {O}_{\psi })$ is again the canonical weakly equivariant local system attaching ${\text {HP}}_0(\mathcal {O}(S_x))$ to each point $x \in \mathcal {O}_{\psi }$.

By Theorem 4.4, $K_{\mathcal {O}_\psi } = \bigoplus _{\mathcal {O}_\chi =\mathcal {O}_\psi } V^*_\chi \otimes L_\chi$. Applying the two paragraphs above, we conclude that $K_{S_\phi \cap \mathcal {O}_\chi } = \bigoplus _{\mathcal {O}_\tau = \mathcal {O}_\chi } V^*_\tau [\dim (S_\phi \cap \mathcal {O}_\chi )-\dim \mathcal {O}_\chi ] \otimes L_\tau |_{S_\phi \cap \mathcal {O}_{\chi }}$. But $\dim (S_\phi \cap \mathcal {O}_\chi )- \dim \mathcal {O}_{\chi } = -\dim \mathcal {O}_\phi$. Summing over all $\chi$ yields the proposition.

Now, pushing forward $M(S_\phi \cap \mathcal {N})$ to a point, we obtain Corollary 2.5.

## Hamiltonian reduction and a mirabolic generalization

### An alternative Proof of Theorem 4.4

We sketch an alternative way to complete the proof of Theorem 4.4, using the functor of Hamiltonian reduction. The details, which are easily checked, are left to the interested reader. Let *G* be a connected complex Lie or algebraic group with $\mathfrak {g}= {\text {Lie}}G$. The Harish-Chandra homomorphism is a surjective morphism $\delta : \mathcal {D}(\mathfrak {g}^*)^G \rightarrow \mathcal {D}(\mathfrak {h}^*)^W$. This descends to an isomorphism $({\text {ad}}(\mathfrak {g}) \mathcal {D}(\mathfrak {g}^*))^G {\setminus } \mathcal {D}(\mathfrak {g}^*)^G \mathop {\longrightarrow }\limits ^{\sim } \mathcal {D}(\mathfrak {h}^*)^W$. This allows one to define the functor of Hamiltonian reduction $\widetilde{\mathbb {H}} : \mathrm {mod}$-$(\mathcal {D}(\mathfrak {g}^*),G) \rightarrow \mathrm {mod}$-$\mathcal {D}(\mathfrak {h}^*)^W$, $\widetilde{\mathbb {H}}(M) = M^G$, from the category of finitely generated, strongly *G*-equivariant right $\mathcal {D}(\mathfrak {g}^*)$-modules to the category of finitely generated right $\mathcal {D}(\mathfrak {h}^*)^W$-modules.

We are interested in weakly $\mathbf {C}^\times$-equivariant modules for the squares of the dilation actions on $\mathfrak {g}^*$ and $\mathfrak {h}^*$. Precisely, in the former case we consider weakly $\mathbf {C}^\times$-equivariant, strongly *G*-equivariant right $\mathcal {D}$-modules on $\mathfrak {g}^*$, and in the latter case we consider graded right $\mathcal {D}(\mathfrak {h}^*)^W$-modules. For brevity we will call these weakly equivariant modules on $\mathfrak {g}^*$ or $\mathfrak {h}^*$. Let ${\text {Eu}}_{\mathfrak {g}^*}$ and ${\text {Eu}}_{\mathfrak {h}^*}$ denote the Euler vector fields on $\mathfrak {g}^*$ and $\mathfrak {h}^*$, so that the square of the dilation action in question is generated by twice the Euler vector field.

One calculates that $\delta ({\text {Eu}}_{\mathfrak {g}^*}) = {\text {Eu}}_{\mathfrak {h}^*} - N$ where $N = |R^+|$ and $R^+$ a choice of positive roots for *W*. Therefore, the functor $\widetilde{\mathbb {H}}$ induces a functor between weakly $\mathbf {C}^\times$-equivariant modules such that $\widetilde{\mathbb {H}}(M)(2N)$ is a strongly $\mathbf {C}^\times$-equivariant $\mathcal {D}(\mathfrak {h}^*)^W$-module if *M* is a strongly $\mathbf {C}^\times$-equivariant $\mathcal {D}(\mathfrak {g}^*)$-module, where as before $M(k) := M \otimes _\mathbf {C}\mathbf {C}_{-k}$.

We have

6.1 $$\begin{aligned} \widetilde{\mathbb {H}}(M(\mathcal {N})) = \mathcal {O}(\mathfrak {h}^*)^W_+ \mathcal {D}(\mathfrak {h}^*)^W {\setminus } \mathcal {D}(\mathfrak {h}^*)^W . \end{aligned}$$

The rings $\mathcal {D}(\mathfrak {h}^*)^W$ and $\mathcal {D}(\mathfrak {h}^*) \rtimes \mathbf {C}[W]$ are Morita equivalent, compatibly with weak equivariance. Let $\mathbb {H}$ be the composite functor $\mathrm {mod}$-$(\mathcal {D}(\mathfrak {g}^*),G) \rightarrow \mathrm {mod}$-$\mathcal {D}(\mathfrak {h}^*) \rtimes \mathbf {C}[W]$. Then

$$\begin{aligned} \mathbb {H}(M(\mathcal {N})) \simeq \mathcal {O}(\mathfrak {h}^*)^W_+ {\setminus } \mathcal {O}(\mathfrak {h}^*) \otimes _{\mathcal {O}(\mathfrak {h}^*) \rtimes \mathbf {C}[W]} \mathcal {D}(\mathfrak {h}^*) \rtimes \mathbf {C}[W]. \end{aligned}$$

The $\mathcal {D}(\mathfrak {h}^*) \rtimes \mathbf {C}[W]$-modules $\varDelta (\chi ) := \chi \otimes _{\mathcal {O}(\mathfrak {h}^*) \rtimes \mathbf {C}[W]}(\mathcal {D}(\mathfrak {h}^*) \rtimes \mathbf {C}[W])$, for $\chi \in \mathrm {Irr} (W)$, are simple and $\mathbf {C}^\times$-equivariant, where $\mathcal {O}(\mathfrak {h}^*)_+$ acts by zero on $\chi$ and the group $\mathbf {C}^{\times }$ acts trivially on $\chi$. It has been shown in [12] and [15], respectively, that $M(\mathcal {N})$ is semisimple and that $\mathbb {H}({\text {IC}}(\mathcal {O}_\chi ,L_\chi )) \ne 0$ for all simple summands ${\text {IC}}(\mathcal {O}_\chi ,L_\chi )$ of $M(\mathcal {N})$.^1^ Since $\mathbb {H}$ is a quotient functor, $\mathbb {H}({\text {IC}}(\mathcal {O}_\chi ,L_\chi ))$ is a simple $\mathcal {D}(\mathfrak {h}^*) \rtimes \mathbf {C}[W]$-module. Therefore, $\mathbb {H}({\text {IC}}(\mathcal {O}_\chi ,L_\chi ))(2N) \simeq \varDelta (\lambda )$ for some $\lambda$. The key to proving Theorem 4.4 is to identify $\lambda$. Recall that $\sigma$ denotes the sign representation.

#### Proposition 6.1

As strongly $\mathbf {C}^\times$-equivariant $\mathcal {D}(\mathfrak {h}^*) \rtimes \mathbf {C}[W]$-modules, $\mathbb {H}({\text {IC}}(\mathcal {O}_\chi ,L_\chi ))(2N) \simeq \varDelta (\chi \otimes \sigma )$.

#### Proof

One can check that the functor of Hamiltonian reduction commutes with Fourier transform. Therefore, it suffices to show that $\mathbb {H}({\text {IC}}(\mathcal {O}_\chi ,L_\chi )^F)(2N) \simeq \varDelta ^F(\chi \otimes \sigma )$, where $\varDelta ^F(\chi \otimes \sigma ) := \chi \otimes _{{\text {Sym}}\,\mathfrak {h}^* \rtimes \mathbf {C}[W]}\mathcal {D}(\mathfrak {h}^*) \rtimes \mathbf {C}[W]$. Let $\nu : \widetilde{\mathfrak {g}^*} \rightarrow \mathfrak {g}^*$ be Grothendieck’s simultaneous resolution. Then the direct image $\nu _* \varOmega _{\widetilde{\mathfrak {g}^*}}$ carries an action of *W* such that $\nu _* \varOmega _{\widetilde{\mathfrak {g}^*}} \simeq \bigoplus _{\chi \in \mathrm {Irrep}(W)} \chi \otimes N_{\chi }$ as a $(W,\mathcal {D}(\mathfrak {g}^*))$-bimodule. By [12, Theorem 5.2], $N_{\chi \otimes \sigma } \simeq {\text {IC}}(\mathcal {O}_\chi ,L_\chi )^F$. Thus, it suffices to show that $\mathbb {H}(N_{\chi }) \simeq \varDelta ^F(\chi )$ as $\mathcal {D}(\mathfrak {h}^*) \rtimes W$-modules. If $\mathfrak {g}_{\mathrm {rs}}^*$ denotes the open subset of $\mathfrak {g}^*$ of elements whose image under the Killing form are regular semisimple in $\mathfrak {g}$, then $N_{\chi } |_{\mathfrak {g}^*_{\mathrm {rs}}}$ is a *G*-equivariant regular connection on $\mathfrak {g}^*_{\mathrm {rs}}$. Thus, it descends to a regular connection on $\mathfrak {h}_{\mathrm {reg}}^* / W$, whose image under the de Rham functor is a local system $R_{\chi }$. By the Springer correspondence, $R_{\chi }$ is isomorphic to the pullback of $\chi$ along the quotient map $\pi _1 (\mathfrak {h}_{\mathrm {reg}}^* / W) \twoheadrightarrow W$. In more detail, the local system on $\mathfrak {h}_{\mathrm {reg}}^* / W$ corresponding to $\nu _* \varOmega _{\widetilde{\mathfrak {g}^*}}$ is the pullback of the regular representation under $\pi _1(\mathfrak {h}_{\mathrm {reg}}^*/W) \twoheadrightarrow W$, which is how the action of *W* is defined on $\nu _* \varOmega _{\widetilde{\mathfrak {g}^*}}$ in the first place, and from this the statement follows.

Similarly, both $\mathbb {H}(N_{\chi })$ and $\varDelta ^F(\chi )$ restrict to *W*-equivariant regular connections on $\mathfrak {h}_{\mathrm {reg}}^*$. Therefore, if $e \in \mathbf {C}[W]$ is the trivial idempotent, $e(\mathbb {H}(N_{\chi }) |_{\mathfrak {h}_{\mathrm {reg}}^*})$ and $e( \varDelta ^F(\chi ) |_{\mathfrak {h}_{\mathrm {reg}}^*})$ are regular connections on $\mathfrak {h}_{\mathrm {reg}}^* / W$. It is clear that the de Rham functor applied to $e( \varDelta ^F(\chi ) |_{\mathfrak {h}_{\mathrm {reg}}^*})$ gives the local system $R_{\chi }$. Thus, it suffices to show that the de Rham functor applied to $e(\mathbb {H}(N_{\chi }) |_{\mathfrak {h}_{\mathrm {reg}}^*})$ equals $R_{\chi }$. Equivalently, if $\mathbb {H}^{\perp }$ is the right adjoint to $\mathbb {H}$, then it suffices to show that $\mathbb {H}^{\perp }(\varDelta ^F(\chi ) ) |_{\mathfrak {g}^*_{\mathrm {rs}}} \simeq N_{\chi } |_{\mathfrak {g}^*_{\mathrm {rs}}}$ as *G*-equivariant regular connections. This is proved in the same way as [17, Proposition 5.5.1], using all of the preceding.

The graded multiplicity of $\chi$ in $\mathcal {O}(\mathfrak {h}^*)/\mathcal {O}(\mathfrak {h}^*)^W_+$ is given by the graded vector space $V_{\chi }$ with Hilbert series $K_{\mathfrak {g},\chi }(y^2)$ and

6.2 $$\begin{aligned} \mathcal {O}(\mathfrak {h}^*)^W_+ {\setminus } \mathcal {O}(\mathfrak {h}^*) \otimes _{\mathcal {O}(\mathfrak {h}^*) \rtimes \mathbf {C}[W] } \mathcal {D}(\mathfrak {h}^*)\rtimes \mathbf {C}[W] = \bigoplus _{\chi \in \mathrm {Irrep} (W)} V_{\chi } \otimes \varDelta (\chi ) \end{aligned}$$

as weakly equivariant modules. Therefore, if $U_{\chi }$ is the graded multiplicity of ${\text {IC}}(\mathcal {O}_\chi ,L_\chi )$ in $M(\mathcal {N})$, Eqs. (6.1) and (6.2) imply that $U_{\chi } \simeq V_{\chi \otimes \sigma }(2N)$. The socle of the Gorenstein ring $\mathcal {O}(\mathfrak {h}^*)/\mathcal {O}(\mathfrak {h}^*)^W_+$ is in degree 2*N*, where it carries a copy of the sign representation. Therefore, $\mathcal {O}(\mathfrak {h}^*)/\mathcal {O}(\mathfrak {h}^*)^W_+$ is, up to a shift by $-2N$ and twisting by $\sigma$, isomorphic as a graded *W*-module to its dual. This implies that $V_{\chi \otimes \sigma }(2N) \simeq V_{\chi }^*$ as graded vector spaces, and hence $h(U_{\chi };y^2) = h(V_{\chi }^*;y^2) = K_{\mathfrak {g},\chi }(y^{-2})$.

### The mirabolic case

In this section only, let $\mathfrak {g}= \mathfrak {gl}(V)$ for some *n*-dimensional vector space *V* and $G = GL(V)$. Then *G* acts diagonally on $\mathfrak {g}^* \times V$. The group $\mathbb {C}^{\times }$ also acts by dilations *along the* $\mathfrak {g}^*$ *factor*, i.e., $\alpha \cdot (X, v) = (\alpha ^{-2} X, v)$. Fix $c \in \mathbf {C}$, thought of as a character $X \mapsto c \mathrm {Tr}(X)$ of $\mathfrak {g}$. If $\mu : \mathfrak {g}\rightarrow \mathcal {D}$ is the quantum moment map, then a right $\mathcal {D}$-module *M* is said to be (*G*, *c*)-monodromic if $\mu _M(X) + \mu (X) = c \cdot {\text {Id}}_M$, where $\mu _M$ is the differential of the *G* action on *M*. (We have a sum rather than a difference because we are considering right $\mathcal {D}$-modules.) Similarly, we say that *M* is $(\mathbf {C}^{\times },d)$-monodromic if $2 {\text {Eu}}_M + 2 {\text {Eu}}_{\mathfrak {g}^*} = d \cdot {\text {Id}}_M$, where ${\text {Eu}}_M$ is the differential of the $\mathbf {C}^{\times }$ action on *M*. These notions can be defined sheaf theoretically; see [2, Definition 2.3.2]. Note that monodromic $\mathcal {D}$-modules are automatically weakly equivariant.

The analogue of the $\mathcal {D}$-module $M(\mathcal {N})$ in this case is the mirabolic Harish-Chandra $\mathcal {D}$-module (or rather its Fourier transform), $M_c(\mathcal {N}\times V)$, as introduced in [9]. As a (*G*, *c*)-monodromic, weakly equivariant $\mathcal {D}$-module on $\mathfrak {g}\times V$,

$$\begin{aligned} M_c(\mathcal {N}\times V) = (\mathcal {O}(\mathfrak {g}^*)_+^G \ \mathcal {D}+ \mu _c(\mathfrak {g}) \ \mathcal {D}) {\setminus } \mathcal {D}. \end{aligned}$$

where $\mu _c(\mathfrak {g}) = \{ \mu (X) - c \mathrm {Tr}(X) \ | \ X \in \mathfrak {g}\}$. This $\mathcal {D}$-module can also be viewed as the natural module associated with the *c*-twisted action of $\mathfrak {g}$ on $\mathcal {N}\times V$, as in [7, Remark 2.17].

The space $\mathcal {N}\times V$ consists of finitely many *G*-orbits. The orbits with fundamental group $\mathbf {Z}$ are naturally labeled by partitions $\lambda \in \mathcal {P}_n$ of *n*. Each of these orbits $\mathcal {O}_{\lambda }$ admits a unique irreducible (one-dimensional) (*G*, *c*)-monodromic local system $L_{\lambda ,c}$. For each $\lambda \in \mathcal {P}_n$, set $c_{\lambda } := c (n_{\lambda ^t} - n_{\lambda })$, where $\lambda ^t$ the dual of $\lambda$; recall that $n_{\lambda } = \sum _i (i-1) \lambda _i$ is the partition statistic.

We will say that *c* is *generic* if $c \notin \{\frac{r}{m} \mid 1 \le m \le n, \gcd (r,m)=1\}$.

#### Theorem 6.2

Assume *c* is generic. There exists a permutation $\tau$ of $\mathcal {P}_n$ such that $c_{\tau (\lambda )} = c_{\lambda }$ and an isomorphism of (*G*, *c*)-monodromic, weakly $\mathbf {C}^{\times }$-equivariant $\mathcal {D}$-modules

$$\begin{aligned} M_c(\mathcal {N}\times V) \simeq \bigoplus _{\lambda \vdash n} V_{\tau (\lambda )}^* \otimes \mathrm {IC}(\mathcal {O}_{\lambda },L_{\lambda ,c}), \end{aligned}$$

where $V_\lambda ^*$ is a weight-graded vector space with Hilbert series $K_{\mathfrak {g},\lambda }(y^{-2})$.

The basic idea behind the proof of this theorem is essentially the same as the one outlined in Sect. 6.1. Again, there is a functor of Hamiltonian reduction, $\mathbb {H}_c : \mathscr {C}_c \rightarrow \mathcal {O}_{-c}$, where $\mathscr {C}_c$ is the category of (*G*, *c*)-monodromic $\mathcal {D}$-modules supported on $\mathcal {N}\times V$ and $\mathcal {O}_{c}$ denotes category $\mathcal {O}$ for the rational Cherednik algebra $H_c(S_n)$; see [11] for details. In the case where *c* is generic, category $\mathcal {O}_c$ for the rational Cherednik algebra is semisimple and the functor of Hamiltonian reduction induces an equivalence between $\mathscr {C}_c$ and category $\mathcal {O}_{-c}$ (see [1, Proposition 9.13]). The argument of Sect. 6.1 is applicable in this setting, though it is more involved since the $\mathcal {D}$-modules $\mathrm {IC}(\mathcal {O}_{\lambda },L_{\lambda ,c})$ are $(\mathbf {C}^{\times },c_{\lambda })$-monodromic, unlike the classical setting where they can be endowed with a $\mathbf {C}^{\times }$-equivariant structure. Moreover, the reason for the occurrence of the permutation $\tau$ is that the analogue of Proposition 6.1 is missing in this context. This is because the key to the proof of Proposition 6.1 is the geometric construction of the simple modules $N_{\chi }$. Since *c* is assumed to be generic, there is no analogous construction for the corresponding simple mirabolic modules. It is an interesting question if $\tau$ is the identity, and if not, it would be interesting to compute it. (There would not seem to be an obvious nontrivial permutation satisfying $c_{\tau (\lambda )}=c_\lambda$.)

As shown in [2], the case where *c* is not generic is much more interesting. (In particular, there the category of mirabolic sheaves need not be semisimple, and we expect $M_c(\mathcal {N}\times V)$ not to be semisimple when the category is not.) We will return to this in future work, where details of the proof of Theorem 6.2 will also be given.

## Footnotes

1 In [15] both statements are proved without explicitly using the proof of [12] of the first statement.

## References

[1] Bellamy, G., Boos, M.: The (cyclic) enhanced nilpotent cone via quiver representations (2016). arXiv:1609.04525v2

[2] Bellamy G, Ginzburg V (2015). Hamiltonian reduction and nearby cycles for mirabolic $$\fancyscript {D}$$-modules. Adv. Math..

[3] Braden, T., Licata, A., Proudfoot, N., Webster, B.: Quantizations of conical symplectic resolutions II: category O (2014). arXiv:1407.0964

[4] Etingof, P., Schedler, T.: Poisson traces, D-modules, and symplectic resolutions (2017). arXiv:1705.00423 10.1007/s11005-017-1024-1 PMC5818674 29497236

[5] Etingof P, Schedler T (2010). Poisson traces and $${\cal{D}} $$-modules on Poisson varieties. Geom. Funct. Anal..

[6] Etingof P, Schedler T (2010). Traces on finite $${\cal{W}}$$-algebras. Transform. Groups.

[7] Etingof, P., Schedler, T.: Coinvariants of Lie algebras of vector fields on algebraic varieties (2012). arXiv:1211.1883 **(to appear in Asian J. Math.)**

[8] Etingof, P., Schedler, T.: Poisson traces for symmetric powers of symplectic varieties. Int. Math. Res. Not. (2013). doi:10.1093/imrn/rnt031; arXiv:1109.4712

[9] Finkelberg M, Ginzburg V (2010). On mirabolic $$\fancyscript {D}$$-modules. Int. Math. Res. Not. IMRN.

[10] Gan WL, Ginzburg V (2002). Quantization of Slodowy slices. Int. Math. Res. Not..

[11] Gan, W.L., Ginzburg, V.: Almost-commuting variety, $$\fancyscript {D}$$-modules, and Cherednik algebras. IMRP Int. Math. Res. Pap. 26439, 1–54 (2006) **(with an appendix by Ginzburg)**

[12] Hotta R, Kashiwara M (1984). The invariant holonomic system on a semisimple Lie algebra. Invent. Math..

[13] Losev, I.: Finite W-algebras (2010). arXiv:1003.5811

[14] Levasseur T, Stafford JT (1996). The kernel of an homomorphism of Harish-Chandra. Ann. Sci. École Norm. Sup. (4).

[15] Levasseur T, Stafford JT (1997). Semi-simplicity of invariant holonomic systems on a reductive Lie algebra. Am. J. Math..

[16] Lusztig G (1981). Green polynomials and singularities of unipotent classes. Adv. Math..

[17] McGerty K (2012). Microlocal $$KZ$$-functors and rational Cherednik algebras. Duke Math. J..

[18] Proudfoot, N.: Hypertoric Poisson homology in degree zero. Algebr. Geom (2014). arXiv:1210.2082

[19] Proudfoot, N., Schedler, T.: Poisson-de Rham homology of hypertoric varieties and nilpotent cones. Sel. Math. (2016). doi:10.1007/s00029-016-0232-3; arXiv:1405.0743

